# Developing a predictive risk model for first-line antiretroviral therapy failure in South Africa

**DOI:** 10.7448/IAS.19.1.20987

**Published:** 2016-09-26

**Authors:** Julia K Rohr, Prudence Ive, C Robert Horsburgh, Rebecca Berhanu, Kate Shearer, Mhairi Maskew, Lawrence Long, Ian Sanne, Jean Bassett, Osman Ebrahim, Matthew P Fox

**Affiliations:** 1 Center for Global Health & Development, Boston University, Boston, MA, USA; 2 Clinical HIV Research Unit, Department of Internal Medicine, School of Clinical Medicine, Faculty of Health Sciences, University of the Witwatersrand, Johannesburg, South Africa; 3 Department of Epidemiology, Boston University School of Public Health, Boston, MA, USA; 4 Health Economics and Epidemiology Research Office, Department of Internal Medicine, School of Clinical Medicine, Faculty of Health Sciences, University of the Witwatersrand, Johannesburg, South Africa; 5 Right to Care, HIV Testing Center, Johannesburg, South Africa; 6 Witkoppen Health and Welfare Centre, Johannesburg, South Africa; 7 Department of Medical Microbiology, University of Pretoria, Pretoria, South Africa

**Keywords:** antiretroviral therapy, predictive model, prognostic score, treatment failure, South Africa, resource-limited settings, public health

## Abstract

**Introduction:**

A substantial number of patients with HIV in South Africa have failed first-line antiretroviral therapy (ART). Although individual predictors of first-line ART failure have been identified, few studies in resource-limited settings have been large enough for predictive modelling. Understanding the absolute risk of first-line failure is useful for patient monitoring and for effectively targeting limited resources for second-line ART. We developed a predictive model to identify patients at the greatest risk of virologic failure on first-line ART, and to estimate the proportion of patients needing second-line ART over five years on treatment.

**Methods:**

A cohort of patients aged ≥18 years from nine South African HIV clinics on first-line ART for at least six months were included. Viral load measurements and baseline predictors were obtained from medical records. We used stepwise selection of predictors in accelerated failure-time models to predict virologic failure on first-line ART (two consecutive viral load levels >1000 copies/mL). Multiple imputations were used to assign missing baseline variables. The final model was selected using internal-external cross-validation maximizing model calibration at five years on ART, and model discrimination, measured using Harrell's C-statistic. Model covariates were used to create a predictive score for risk group of ART failure.

**Results:**

A total of 72,181 patients were included in the analysis, with an average of 21.5 months (IQR: 8.8–41.5) of follow-up time on first-line ART. The final predictive model had a Weibull distribution and the final predictors of virologic failure were men of all ages, young women, nevirapine use in first-line regimen, low baseline CD4 count, high mean corpuscular volume, low haemoglobin, history of TB and missed visits during the first six months on ART. About 24.4% of patients in the highest quintile and 9.4% of patients in the lowest quintile of risk were predicted to experience treatment failure over five years on ART.

**Conclusions:**

Age, sex, CD4 count and having any missed visits during the first six months on ART were the strongest predictors of ART failure. The predictive model identified patients at high risk of failure, and the predicted failure rates over five years closely reflected actual rates of failure.

## Introduction

South Africa has the world's largest HIV epidemic, with approximately 6.8 million people living with HIV in 2014 [1]. Over a period of 10 years after South Africa launched its national HIV programme in 2004, a substantial number of patients have failed first-line and require second-line antiretroviral therapy (ART). Clinical studies from South Africa show that after five years on treatment, approximately 14% of patients on first-line ART experience virologic failure [2]. Individual predictors of first-line failure have been identified, such as CD4 count, sex, age, clinic attendance, ART adherence and general health [2–10], yet few studies in resource-limited settings have been large enough to model the interactions between factors necessary for appropriate predictive modelling, and the relative impact of each predictor has not been thoroughly investigated.

The aims of this study are to estimate absolute first-line ART failure risk over five years on treatment as a function of a baseline profile of demographic, clinical and immunologic factors and their interactions, and to develop a predictive model that can be applied to other South African clinic populations, giving estimates of proportion of patients needing second-line ART over time. At the population level, this model can provide long-term estimates of the need for second-line ART in South Africa over five years for patients who remain in care, given characteristics of the population beginning ART. At the individual level, this model provides risk groups for treatment failure for patients who begin treatment, which can be used to identify patients at the highest risk. Identifying high-risk patients allows for targeted adherence interventions and counselling, ideally avoiding treatment failure and the need for second-line, or potentially third-line ART, and more powerful drugs.

## Methods

### Data source and study population

We conducted an observational cohort study using routinely collected medical record data from the Right to Care clinical HIV cohort, which includes patients who had initiated treatment between 2004 and 2013 from nine HIV clinics in South Africa that follow national treatment guidelines (seven in Gauteng and two in the Mpumalanga Province). Clinics used an electronic medical record system that records basic information on demographics (e.g. date of birth, sex), clinical information (e.g. height, weight), date of visits, lab results, diagnoses and HIV treatment. We labelled each clinic letter A through I. Retrospective analysis of the Right to Care cohort was approved by the Human Research Ethics Committee of the University of the Witwatersrand. Boston University provided permission for analysis of de-identified data.

We included treatment-naïve adult patients (aged ≥18 years) initiating standard first-line HIV treatment who had received at least six months of ART. A standard first-line regimen was defined as two nucleoside reverse transcriptase inhibitors (NRTIs) (stavudine (d4T), zidovudine (AZT) or tenofovir (TDF) plus lamivudine (3TC) or emtricitabine (FTC)) and one non-nucleoside reverse transcriptase inhibitor (NNRTI) (either efavirenz (EFV) or nevirapine (NVP)).

### Study variables

#### Predictors

Predictor variables came from clinic data at ART initiation, including age, sex, year of ART initiation, clinic, CD4 count, World Health Organization (WHO) stage, body mass index (BMI), haemoglobin, total lymphocytes, mean corpuscular volume (MCV), red blood cell count (RBC), creatinine, creatinine clearance, alanine transaminase (ALT), aspartate transaminase (AST), blood pressure, history of tuberculosis, peripheral neuropathy, NRTIs and NNRTI in first-line regimen, history of alcohol and history of smoking. Having any missed visits by more than seven days prior to our start of follow-up at six months on ART was also included as a predictor. Baseline lab measures were taken from the test closest to ART initiation, using values 90 days before to seven days after ART initiation.

#### Outcomes

Follow-up time began at six months after ART initiation at the time of the first scheduled viral load. If patients had an earlier viral load measurement (at three to six months on ART), follow-up time began at the time of viral load measurement for these individuals. Our primary outcome was virologic failure after first-line initiation. Date of virologic failure was defined as the date of the second of the two consecutive viral load measurements >1000 copies/mL. Person-time ended on the occurrence of any of the following: first-line treatment failure, switch to second-line ART (defined as switch to a protease inhibitor (PI)-based regimen plus addition of at least one new NRTI), loss to follow-up, death, transfer to another clinic or date of dataset closure (April 2014). Loss to follow-up was defined as not visiting the clinic within six months of closure of the study, and the date of loss to follow-up was the date of the last visit.

### Statistical methods

#### Model development

Distributions of all baseline variables were examined in summary and stratified by the year of ART initiation. The overall failure rate and rates by clinic were examined with Kaplan-Meier curves. The predictive model for ART failure was developed using validation of accelerated failure-time survival models, since these parametric models allow for direct calculation of predicted probabilities of survival at specific time points [11,12]. First, unadjusted models were run for each predictor. Akaike's information criteria (AIC) scores were used to compare continuous variables and categorizations of variables to find the optimal version of each predictor. Unadjusted models were run using all data, and also stratified by clinic, to look for site-specific predictors. Year and clinic were investigated in unadjusted models but not in the final model in order to make the model applicable for future use and outside clinics. Models with estimates adjusted for year and clinic were later considered in the final candidate model to determine if adjustments would improve predictive value.

Candidate multivariate predictive models were developed using stepwise selection with a value of *p*<0.2, and later evaluated for predictive value. Biologically plausible multiplicative interactions between predictors with *p*<0.2 were considered in final candidate models. Multiple imputation was used for missing predictor variable data [13,14]. Variables with >50% baseline values missing were not used [15]. The imputation model included all baseline variables that were possible predictors, as well as the indicators for failure, loss to follow-up, switch to second-line ART, transfer to another clinic, or death, and a variable for time to failure. No outcome variables were imputed. Seven dataset imputations were created and were combined using the proc mianalyze procedure in SAS.

#### Model fit

The best candidate predictive models were selected based on several model fit measures. AIC scores assessed overall model fit. In addition, statistics to measure model discrimination and calibration were calculated in a cross-validation procedure. Cross-validation was used to ensure high predictive value of the models, since the model giving the best prediction on data used to develop it may not have the best performance on independent data [11,16,17].

We used internal-external cross-validation (IECV), where each candidate predictive model was developed on all clinics excluding one, and model performance was tested on independent data using the excluded clinic [11]. IECV was repeated nine times to test performance in all nine clinics, and average model discrimination and calibration statistics weighted by the clinic population were calculated. Harrell's C-statistic, a survival analysis approximation to the C-statistic which measures the proportion of all subject pairs where prediction of an earlier time to event is consistent with actual outcomes, was used as the measure of model discrimination [11,18,19]. Predicted five-year survival was compared with actual five-year survival in the IECV procedure to assess model calibration. When selecting the best predictive model, clinical validity (results from IECV) was prioritized over statistical validity (AIC score), and discrimination (C-statistic) was prioritized over model calibration (difference between actual and predicted five-year survival).

The final model was used to assess the impact of individual baseline predictors and interactions between predictors on absolute risk of treatment failure. Since model discrimination is especially relevant for those who initiated care more recently, we also evaluated the C-statistic for the subset of the population that initiated ART in recent years (2011 and later). Outcomes from this model include scores to calculate a failure risk group based on an individual's baseline variables, and calculation of absolute risk of failure at one through five years on ART based on an individual's baseline variables.

#### Risk score

Scoring for a predicted risk group was directly generated from beta estimates of the final predictive model. A survival estimate can be calculated from the Weibull model at any time point based on a transformation of the summation of beta estimates *S*(*t*)*=exp*{*−*[t*exp(*−sum*(*βx*))]^(1/*s*)}, where *t*=time and *s*=the scale parameter. We used the summation of beta estimates for each individual to determine the relative likelihood of failure, and created risk groups based on quintiles of these scores. To simplify risk group calculation for use in clinics, we transformed the beta estimates for all predictors by setting the lowest risk group as the reference category, removing the intercept (baseline hazard), multiplying each beta estimate by 10 and rounding to the nearest digit. This transformation created a whole number for each predictor that would allow summation of all risk factors to calculate a risk score.

### Sensitivity analyses

First-line failure could not occur if death or switch to second-line ART occurred first. The impact of these competing risks was assessed graphically [20]. Patients who remained in care but had a reasonable chance of having been misclassified as non-failures, such as patients who experience death in care without documented treatment failure, and patients who switched to second-line ART before documented failure, were considered to have treatment failure in a sensitivity analysis. In addition, to investigate the impact of missing viral loads, a sensitivity analysis was performed in which person-time was defined as time in treatment with regular viral load measurements, with patients without a viral load for more than one year being censored. Lastly, analyses excluding clinics with much higher or lower rates of failure than average were also performed.

## Results

### Study sample

There were 72,181 adult patients who initiated a standard first-line ART regimen after 2003, and had at least six months of follow-up. Most were aged between 30 and 39 years (43.7%), female (65.0%) and had a first-line ART regimen containing d4T (60.0%) and EFV (86.9%). Follow-up time ended in treatment failure for 9.5% of patients, death for 4.8%, switch to second-line ART for 3.5%, loss to follow-up for 19.7%, clinic transfer for 25.1% and censoring for the remaining 37.5% at the end of the study period. Median follow-up time was 21.5 months (IQR: 8.8–41.5). After 5.5 years on treatment, >15% of subjects experienced first-line treatment failure. The failure rate was similar in all clinics except for Clinic E, which showed a much higher rate, and Clinic I, which had a lower rate.

### Variable frequencies

Values of potential predictor variables by the year of ART initiation are shown in Table 1. TDF became more commonly prescribed over d4T starting in 2011, as guidelines changed. EFV was prescribed more often than NVP, especially in recent years. Patients initiating treatment became slightly healthier over time, with decreasing TB (25.1% in 2004, 8.2% in 2013), higher initiating CD4 counts (22.4% <50 cells/mm^3^ in 2004, 12.4% in 2013) and fewer underweight patients in later years (12.2% in 2004, 7.7% in 2013). Visit adherence worsened over time, with 6.0% having missed visits in the first six months on treatment in 2004 and 35.4% in 2013. Most patients had five to six scheduled visits, and 84% who missed visits missed only one visit.

**Table 1:** Baseline characteristics of the study population stratified by year of ART initiation

	2004	2005	2006	2007	2008	2009	2010	2011	2012	2013
N	2025	3998	6616	7298	9535	11,361	11,312	8820	7314	3902
	%	%	%	%	%	%	%	%	%	%
Female	68.5	68.1	65.3	66.0	66.5	63.6	65.5	63.2	64.1	61.7
Age (years)										
18–24.9	4.7	5.4	5.6	5.9	6.0	5.9	6.4	5.9	6.8	7.4
25–29.9	17.3	16.6	14.8	15.8	15.6	15.6	16.2	16.0	16.2	18.1
30–34.9	25.3	24.7	24.3	24.1	22.3	22.4	21.8	21.7	21.8	21.8
35–39.9	21.2	21.3	20.5	21.2	21.4	20.6	20.9	21.5	20.9	20.3
40–44.9	14.4	15.0	16.0	14.6	14.8	14.6	14.6	14.6	14.3	13.3
45–49.9	9.2	8.8	9.4	9.1	9.5	10.2	10.0	9.7	9.3	9.4
50–54.9	4.9	4.9	5.4	5.5	5.6	5.6	5.4	5.7	5.7	5.2
≥55	3.0	3.2	3.9	3.8	4.8	5.0	4.7	4.9	5.0	4.6
TB positive	25.1	21.7	14.4	12.1	12.4	18.2	17.4	13.9	9.2	8.2
NRTI in first-line ART										
TDF	0.7	0.9	1.5	3.1	4.7	5.5	49.6	85.5	86.3	92.5
AZT	8.4	6.7	6.5	7.1	7.4	6.0	6.9	4.3	4.4	3.3
D4T	90.9	92.4	92.0	89.8	87.9	88.5	43.5	10.2	9.3	4.2
NNRTI in first-line ART										
EFV	86.7	88.0	87.0	83.4	80.3	84.8	87.2	89.4	92.7	96.7
NVP	13.3	12.0	13.0	16.6	19.7	15.2	12.8	10.6	7.3	3.3
Missed visits in the first six months on treatment	6.0	10.1	15.6	19.0	20.4	22.8	25.6	26.5	33.8	35.4
WHO stage										
Stage 1	63.1	63.9	68.0	66.5	66.5	70.5	73.4	77.9	83.1	84.8
Stage 2	1.6	1.5	1.5	1.6	2.7	1.4	0.9	0.5	0.6	0.5
Stage 3	31.4	29.5	23.3	22.6	22.4	23.4	21.6	18.0	13.7	12.9
Stage 4	4.0	5.2	7.3	9.3	8.4	4.7	4.1	3.6	2.6	1.7
BMI (kg/m^2^)										
<18.5	12.2	13.6	12.0	12.5	11.2	12.2	10.9	9.1	8.0	7.7
18.5–24.9	41.4	41.2	38.7	38.0	42.7	46.3	42.4	42.6	39.5	39.6
25–29.9	11.6	10.9	10.7	11.9	13.8	15.0	16.2	17.3	18.3	18.5
≥30	5.4	4.3	4.5	5.2	6.8	6.8	9.0	10.0	12.3	11.6
Missing	29.5	30.1	34.1	32.5	25.5	19.8	21.6	21.1	21.9	22.6
CD4 count (cells/mm^3^)										
0–24	12.1	13.3	12.9	11.5	10.6	8.5	8.5	7.5	6.3	6.7
25–49	10.3	9.6	8.8	8.9	8.5	7.7	7.1	6.4	5.2	5.7
50–99	18.5	13.9	14.7	15.3	14.9	14.7	13.5	12.0	9.0	10.1
100–199	21.6	27.0	25.3	29.6	31.1	30.1	27.9	24.0	19.8	20.9
200–349	4.4	5.3	5.6	7.5	10.3	12.9	19.1	20.7	28.6	27.8
≥350	1.2	1.3	1.7	2.6	2.7	4.1	3.0	4.2	5.5	9.6
Missing	31.9	29.7	30.9	24.7	21.9	22.0	21.1	25.2	25.7	19.2
Haemoglobin (g/dL)										
<12	37.7	38.1	39.7	40.3	40.9	42.6	43.3	40.1	38.6	37.1
≥12	32.4	28.9	28.0	27.6	28.8	31.0	32.5	35.6	40.6	41.0
Missing	30.0	33.0	32.3	32.1	30.3	26.4	24.2	24.3	20.8	21.9
Creatinine clearance (mL**/**minute**)**										
Normal (≥90)	1.8	3.0	4.5	11.1	24.0	34.7	50.9	56.8	61.6	61.7
Mild (60–89)	1.1	1.9	2.7	5.1	7.8	12.3	9.7	10.3	9.5	8.7
Moderate (30–59)	0.5	0.9	1.0	1.2	1.8	2.9	2.5	2.6	2.5	2.3
Severe (<30)	0.4	0.3	0.5	0.3	0.6	0.7	1.5	0.8	0.8	1.1
Missing	96.3	94.0	91.3	82.3	65.8	49.5	35.4	29.6	25.7	26.4
Blood pressure										
Low	1.0	0.6	0.7	0.9	1.0	0.8	1.0	0.8	0.7	0.7
Normal	23.3	23.6	22.8	24.9	29.0	32.2	29.8	26.1	28.3	30.8
Borderline high	19.2	17.2	17.6	18.9	22.8	28.2	28.1	29.5	31.6	33.0
Stage 1 hypertension	7.1	6.7	7.2	7.0	10.2	13.6	14.9	18.5	17.8	15.9
Stage 2 hypertension	3.5	3.9	4.3	4.0	4.6	7.2	8.0	10.7	9.7	8.4
Missing	46.0	48.1	47.4	44.4	32.4	18.0	18.1	14.5	11.8	11.2

BMI: body mass index; NNRTI: non-nucleoside reverse transcriptase inhibitor; NRTI: nucleoside reverse transcriptase inhibitor.

### Multivariate adjusted failure-time models

Fit diagnostic graphs and Cox-Snell residuals indicated the Weibull model fit the data best. The final predictors from stepwise selection of predictors in the non-imputed model included age, sex, missed visits during the first six months on treatment, TB history, NNRTI, CD4, MCV and haemoglobin, and interactions between NNRTI and CD4, haemoglobin and CD4, and sex and CD4. When this model was fit to imputed baseline data, all variables still had *p*<0.2 and hazard ratios remained the same. When other predictors and interactions were added back into the model using imputed data, blood pressure and interactions between age and sex, sex and haemoglobin and sex and NNRTI had *p*<0.2. These model variations were considered in IECV.

### Cross-validation of models

We tested 12 models. The selected model (Table 2) had the highest Harrell's C-statistic (60.1) and one of the lowest differences in five-year survival prediction (<0.01% difference between overall actual and predicted failure at five years on ART). Adding BMI or blood pressure values and adjusting parameters for year and clinic did not improve model performance. The model chosen as the predictive model maximized the C-statistic, but when weaker predictors were removed from the model, we found that CD4 count, age, sex and having missed visits in the first six months on treatment could account for most of the model's discrimination. Without NNRTI, MCV, haemoglobin and history of TB, the C-statistic dropped slightly to 59.7. Among the subset of the population that initiated ART in 2011 and later, the final model was very predictive, with a C-statistic of 64.0.

**Table 2:** Final Weibull model estimates to predict first-line virologic ART failure after six months on treatment

Variable	Weibull model beta estimate (95% confidence limits)	*p*	HR
Intercept	9.97 (9.83, 10.11)	<0.0001	
Age (years)			
18–24.9	−0.45 (−0.58, −0.32)	<0.0001	1.47
25–29.9	−0.23 (−0.33, −0.13)	<0.0001	1.22
30–34.9	Ref.		
35–39.9	0.20 (0.09, 0.31)	0.000	0.84
40–44.9	0.25 (0.12, 0.38)	0.000	0.80
45–49.9	0.34 (0.19, 0.50)	<0.0001	0.74
50–54.9	0.29 (0.10, 0.49)	0.004	0.78
≥55	0.36 (0.13, 0.59)	0.003	0.73
Sex			
Male	−0.14 (−0.26, −0.01)	0.028	1.12
Female	Ref.		
NNRTI			
NVP	−0.33 (−0.41, −0.25)	<0.0001	1.33
EFV	Ref.		
CD4 count (cells/mm^3^)			
0–24	−0.63 (−0.73, −0.54)	<0.0001	1.73
25–49	−0.40 (−0.50, −0.30)	<0.0001	1.41
50–99	−0.26 (−0.35, −0.17)	<0.0001	1.25
100–199	Ref.		
200–349	0.16 (0.05, 0.27)	0.006	0.87
≥350	0.37 (0.15, 0.59)	0.002	0.73
MCV (fL)			
<80	Ref.		
80–95	−0.15 (−0.24, −0.06)	0.002	1.14
≥95	−0.28 (−0.41, −0.14)	0.000	1.27
Haemoglobin (g/dL)			
<12	−0.11 (−0.18, −0.04)	0.001	1.10
≥12	Ref.		
History of TB			
Yes	−0.06 (−0.13, 0.02)	0.131	1.05
No	Ref.		
Missed visits			
Yes	−0.40 (−0.46, −0.33)	<0.0001	1.41
No	Ref.		
Sex and age (years)			
Female & 30–35	Ref.		
Male & 18–25	0.20 (−0.12, 0.53)	0.224	0.84
Male & 25–30	0.15 (−0.05, 0.35)	0.144	0.88
Male & 35–40	−0.14 (−0.31, 0.03)	0.099	1.13
Male & 40–45	−0.19 (−0.38, 0.00)	0.048	1.18
Male & 45–50	−0.33 (−0.55, −0.10)	0.004	1.33
Male & 50–55	−0.12 (−0.41, 0.16)	0.393	1.11
Male & ≥55	−0.17 (−0.49, 0.16)	0.314	1.16
Scale	1.16		

MCV: mean corpuscular volume; NNRTI: non-nucleoside reverse transcriptase inhibitor.

### Survival prediction

The simplified version of the predictive score based on quintiles of the population's predicted risk of failure from the final model is displayed in Table 3. Baseline CD4 count along with sex and age group were the most influential components of the risk score. According to the hazard ratios derived from the model, individuals with CD4 count <25 cells/mm^3^ had a 73% increase in hazards of failure compared with individuals with CD4 counts of 100–199 cells/mm^3^ at ART initiation. Individuals who missed visits in the six months following ART initiation were at a significantly higher risk of failure compared with those who missed no visits (HR=1.41, 95% CI=1.33, 1.49), and individuals on NVP rather than EFV had increased hazards of treatment failure (HR=1.33, 95% CI=1.24, 1.42). MCV, haemoglobin and history of TB were weaker predictors of treatment failure (Table 2).

**Table 3:** Approximation of ART failure risk group score based on final model parameters

Score calculation			Score	Risk group	Predicted risk of failure at five years
1. Sex and age (years):			>17	High	24.4%
Females:	Age:		14–17	Medium-high	18.0%
	18–24.9	+8	12–13	Middle	14.8%
	25–29.9	+6	9–11	Medium-low	12.3%
	30–34.9	+4	0–8	Low	9.4%
	35–39.9	+2			
	40–44.9	+1			
	≥45	+0			
Males:	Age:				
	18–24.9	+7			
	25–29.9	+6			
	30–34.9	+5			
	35–49.9	+4			
	≥50	+3			
2. NNRTI:	NVP	+3			
	EFV	+0			
3. CD4 count	0–24	+10			
(cells/mm^3^):	25–49	+8			
	50–99	+6			
	100–199	+4			
	200–349	+2			
	≥350	+0			
4. MCV (fL):	<80	+0			
	80–95	+1			
	≥95	+3			
5. Haemoglobin	<12	+1			
(g/dL):	≥12	+0			
6. History of TB:	Yes	+1			
	No	+0			
7. Missed visits during the first six months on treatment:	Yes	+4			
	No	+0			

Score is calculated by summation of the seven predictors. Variables are measured at ART initiation except for missed visits, which is measured over the first six months on ART. MCV: mean corpuscular volume; NNRTI: non-nucleoside reverse transcriptase inhibitor.

**Figure 1:**
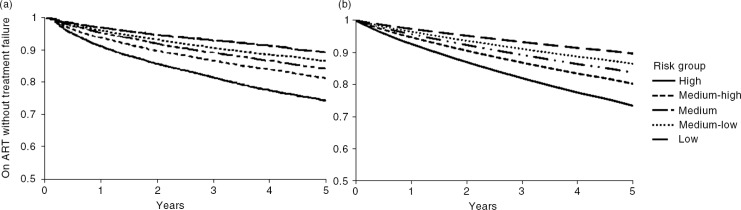
(a) Actual and (b) predicted treatment failure over time past six months on ART by risk group for individuals in study population.

Figure 1a displays actual failure on treatment for individuals in the study population by risk group, compared with model prediction of failure by risk group (Figure 1b). The model predicts that treatment will fail for 24.4% of patients in the high-risk group and 9.4% of patients in the low-risk group in five years’ time (Table 4).

**Table 4:** Cumulative per cent of patients for whom treatment is predicted to fail during the first five years on ART

	Years on ART				
	
Risk group	1	2	3	4	5
High (%)	3.8	10.1	15.4	20.1	24.4
Medium-high (%)	2.7	7.3	11.2	14.7	18.0
Medium (%)	2.2	5.9	9.1	12.1	14.8
Medium-low (%)	1.8	4.9	7.5	10.0	12.3
Low (%)	1.4	3.7	5.7	7.6	9.4

### Sensitivity analyses

When death and switch to second-line were considered as competing risks, patients who died were considered as treatment failures, or patients without a viral load for more than one year were censored there was very little impact on the failure rate estimates. When switches to second-line were considered treatment failures at the date of regimen switch the overall failure rate increased by about 5% over five years.

## Discussion

Through five years on ART, 15% of patients experienced treatment failure, consistent with previous estimates of treatment failure in South Africa [2]. The scale parameter in the Weibull model was slightly greater than one (1.16), indicating that the hazards of failure were fairly constant and suggesting that the need for regular viral load monitoring does not decrease over time.

At a population level, this study goes beyond other study estimates for treatment failure by providing a model for estimating the long-term need for second-line ART among specific populations. For example, the five-year failure rate would drop by 9% if the entire population began treatment at a CD4 count of 200–349 cells/mm^3^ compared with if everyone in the population began treatment at a CD4 count <25 cells/mm^3^, all other factors remaining equal. As CD4 counts at ART initiation increase over time, we expect this will have a positive impact on long-term failure rates. During the time period for this study, guidelines for ART initiation were never above a CD4 threshold of 350 cells/mm^3^, and individuals initiating ART in this CD4 range may qualify for other health issues like TB or high WHO stage. Individuals initiating ART at high CD4 counts with no health complications may encounter even less failure than this model suggests.

At the individual level, this model can be used to identify patients at the highest risk of treatment failure. Clinicians can effectively resort to adherence counselling and behavioural interventions early, before virologic indication of treatment failure. Ideally, interventions would prevent elevated viral loads in patients with a high risk of failure, which would in turn limit accumulation of drug resistance mutations, poor clinical outcomes and the need for more expensive drugs.

### Predictors of treatment failure

CD4 count, sex and age group were the most influential baseline predictors of ART failure, and haemoglobin and history of TB were the least influential. The interaction between age and sex showed that young patients were at the highest risk of failure, particularly young women, but the risk of failure decreased more substantially with age for women than men. Previous studies have shown that being male and of a younger age are associated with higher failure rates [3,4]. Poor visit adherence was also an important predictor of treatment failure and has been consistently associated with poor treatment outcomes [6,7,9,10,21–26]. Factors associated with poor adherence, such as less education, poor financial support, fear of disclosure or stigma, substance abuse and depression should also be considered when identifying patients at risk of failure [27–29].

Other predictors, including low CD4, history of TB, NVP rather than EFV use and low haemoglobin have previously been associated with treatment failure and death [2–5]. Although associations with treatment outcomes and haemoglobin are common, MCV has not previously been shown to predict failure. A model excluding MCV was considered in cross-validation analysis, but inclusion of MCV offered slightly better model discrimination. MCV can become elevated with AZT use over time on treatment, but when the model was stratified by NRTI, the association remained consistent for patients irrespective of their NRTI. Elevated MCV values may reflect macrocytosis caused by food insecurity (folate or vitamin B12 deficiency) or alcohol intake [30].

Although year was not included as a predictor, so that the model may be used in the future, we saw higher failure rates in recent years. The reason for increased failure may be related to poorer patient management and worse visit adherence with more patients in care, or due to differences in laboratory procedures for viral load testing. If trends towards increasing treatment failure continue, the model may under-estimate failure. Similarly, if patient retention improves over time, more treatment failures may occur prior to loss to follow-up, in which case this model would also under-estimate the absolute need for second-line ART.

### Model performance

Cross-validation of candidate models ensured that the chosen model would be most useful with respect to identifying patients at a high risk of failure when applied to external data. Many interaction terms were statistically significant in the model initially but did not offer improved model discrimination or calibration when applied to external data, with the exception of the interaction between age and sex.

The ability of the model to discriminate failure times between individual patients (Harrell's C-statistic) was lower than desired (60%), but the model had good calibration, with a five-year predictive risk equivalent to the actual failure at five years. Risk groups of patients were identified using quintiles of calculated risk of failure in the population, and failure estimates from the model by risk group matched the actual failure by risk group over time.

### Study strengths, weaknesses and future direction

The main weakness of the study is the low discrimination score for the predictive model. The C-statistic of 60% implies that the model correctly identifies a patient is at higher risk than another patient 60% of the time in independent data. Including clinic site could improve model discrimination but could not be done in a predictive model intended for external use. Although not tested outside of South Africa, this model may be applied to other populations with similar demographic and clinical profiles. Future work could identify clinic settings that share predictors of ART failure for developing setting-specific models. The low discrimination score also indicates that treatment failure is a complex event, likely having determinants based on individual behaviour and external circumstances, and therefore cannot be very precisely identified through a model of baseline clinic, lab and demographic variables. Missing data were also a weakness of the study. Multiple imputations were used so that patients with missing predictor variables could be included. Impact of missing or misclassified outcomes was explored in sensitivity analyses.

The main strength of the model is its ability to provide insight into factors associated with the risk of treatment failure and their relative influence, and to identify risk groups of patients most likely to fail. Practically, the identification of high-risk groups for treatment failure has an important application in clinical settings, where high-risk patients could be monitored more closely and receive treatment adherence counselling or other interventions [31]. The large sample size was also a strength of this study and allowed for modelling of interaction terms.

## Conclusions

Our treatment failure model was able to identify patients at risk of failure; estimate the proportion of patients failing treatment over time for those who remain in care; and emphasized the importance of CD4 count, age, sex and visit adherence in determining patients at risk of failure. Future work could expand the model to account for patients who are lost to care, since estimating outcomes for all patients regardless of whether or not they stay in care would be helpful for public health planning. In addition, further research into best practices for ART adherence interventions is an important next step in order to effectively improve outcomes for high-risk patients.
